# Advancements in rapid diagnostics and genotyping of *Piscirickettsia salmonis* using Loop-mediated Isothermal Amplification

**DOI:** 10.3389/fmicb.2024.1392808

**Published:** 2024-09-24

**Authors:** Adolfo Isla, Marcelo Aguilar, Sandra N. Flores-Martin, Claudia A. Barrientos, Genaro Soto-Rauch, Jorge Mancilla-Schulz, Felipe Almendras, Jaime Figueroa, Alejandro J. Yañez

**Affiliations:** ^1^Departamento de Ciencias Básicas, Facultad de Ciencias, Universidad Santo Tomás, Valdivia, Chile; ^2^Escuela de Graduados, Facultad de Ciencias, Universidad Austral de Chile, Valdivia, Chile; ^3^Interdisciplinary Center for Aquaculture Research (INCAR), Universidad de Concepción, Concepción, Chile; ^4^Laboratorio de Biología Molecular de Peces, Instituto de Bioquímica y Microbiología, Facultad de Ciencias, Universidad Austral de Chile, Valdivia, Chile; ^5^Mowi Chile S.A., Puerto Montt, Chile; ^6^Departamento de Investigación y Desarrollo, Greenvolution SpA., Puerto Varas, Chile

**Keywords:** *Piscirickettsia salmonis*, genotyping, Loop-mediated Isothermal Amplification (LAMP), aquaculture, rapid diagnostics, pathogen identification

## Abstract

**Introduction:**

*Piscirickettsia salmonis*, the causative agent of Piscirickettsiosis, poses a significant threat to the Chilean aquaculture industry, resulting in substantial economic losses annually. The pathogen, first identified as specie in 1992, this pathogen was divided into two genogroups: LF-89 and EM-90, associated with different phenotypic mortality and pathogenicity. Traditional genotyping methods, such as multiplex PCR, are effective but limited by their cost, equipment requirements, and the need for specialized expertise.

**Methods:**

This study validates Loop-mediated Isothermal Amplification (LAMP) as a rapid and specific alternative for diagnosing P. salmonis infections. We developed the first qPCR and LAMP assay targeting the species-conserved tonB receptor gene (*ton*B-r, WP_016210144.1) for the specific species-level identification of *P. salmonis*. Additionally, we designed two genotyping LAMP assays to differentiate between the LF-89 and EM-90 genogroups, utilizing the unique coding sequences Nitronate monooxygenase (WP_144420689.1) for LF-89 and Acid phosphatase (WP_016210154.1) for EM-90.

**Results:**

The LAMP assays demonstrated sensitivity and specificity comparable to real-time PCR, with additional benefits including rapid results, lower costs, and simplified operation, making them particularly suitable for field use. Specificity was confirmed by testing against other salmonid pathogens, such as *Renibacterium salmoninarum, Vibrio ordalii, Flavobacterium psychrophilum, Tenacibaculum maritimum*, and *Aeromonas salmonicida*, with no cross-reactivity observed.

**Discussion:**

The visual detection method and precise differentiation between genogroups underscore LAMP's potential as a robust diagnostic tool for aquaculture. This advancement in the specie detection (qPCR and LAMP) and genotyping of *P. salmonis* represents a significant step forward in disease management within the aquaculture industry. The implementation of LAMP promises enhanced disease surveillance, early detection, and improved management strategies, ultimately benefiting the salmonid aquaculture sector.

## 1 Introduction

Piscirickettsiosis, caused by the Gram-negative coccoid bacterium *Piscirickettsia salmonis*, manifests as a systemic infection impacting the Chilean aquaculture industry, resulting in annual losses nearing US$700 million (Maisey et al., 2017; Figueroa et al., 2019). *P. salmonis* was isolated from moribund Coho salmon near Puerto Montt in 1989. This pathogen has become a main disease agent in the 2022 mortality surge among Atlantic salmon, Rainbow trout, and Coho salmon (Sernapesca, 2022; Bravo and Campos, 1989). This pathogen has been described in various regions such as Canada (Otterlei et al., 2016), Norway (Olsen et al., 1997), Scotland and Ireland (Reid et al., 2004).

*P. salmonis* is associated with Salmonid Rickettsial Septicaemia (SRS), a disease that severely affects affecting aquaculture (Brocklebank et al., 1993; Grant et al., 1996; Rodger and Drinan, 1993; Olsen et al., 1997; Rozas and Enriquez, 2014; Mauel et al., 2008). Initially misclassified as an Alphaproteobacteria, *P. salmonis* was later reclassified into the Gammaproteobacteria class through 16S rDNA phylogenetic analysis (Fryer et al., 1992). Further characterization of 16S rDNA from diverse isolates and whole-genome sequencing identified two distinct genetic groups, LF-89 and EM-90, which exhibit significant genetic and pathogenic differences (Heath et al., 2000; Bohle et al., 2014; Bravo and Martinez, 2016; Saavedra et al., 2017; Nourdin-Galindo et al., 2017).

The classification of *P. salmonis* into these genogroups has significantly advanced genotyping methods, leading to the development of Multilocus Sequence Typing (MLST) (Isla et al., 2019) and PCR-Restriction Fragment Length Polymorphism (PCR-RFLP) targeting the 16S rDNA gene (Aravena et al., 2020). MLST has been instrumental in identifying distinct allelic profiles for the LF-89 and EM-90 genogroups, revealing significant genetic diversity and enhancing our understanding of the pathogen's population structure. This method, along with PCR-RFLP, which differentiates these genogroups by analyzing specific patterns in the 16S rDNA gene, has confirmed the genetic distinctions between the groups. While both techniques have greatly contributed to the development of targeted diagnostic tools and epidemiological tracking within aquaculture, they remain resource-intensive and challenging for routine use in standard microbiology laboratories.

A recent Multiplex-PCR strategy based on unique genes identified from 73 fully sequenced genomes has further refined these methods, identifying 1,801 shared coding sequences and unique sequences specific to each genogroup, with 253 sequences in LF-89-like isolates and 291 in EM-90-like isolates (Isla et al., 2021). These advancements have facilitated the development of novel diagnostic methods for distinguishing between genogroups using PCR and qPCR on both *in-vitro* and field isolates (Isla et al., 2021). Despite the efficacy of multiplex PCR for genotyping, its reliance on expensive equipment, specialized lab skills, and susceptibility to primer interference limit its widespread field adoption as a routine diagnostic tool (Soroka et al., 2021; Kreitmann et al., 2023; Ador et al., 2021). Additionally, standardizing this approach may require numerous trials, potentially compromising result accuracy.

Loop-Mediated Isothermal Amplification, or LAMP, is a technique that offers a cost-effective, precise, and rapid approach for various life sciences applications, including pathogen detection and genetic disorder diagnosis (Notomi et al., 2000; Hong et al., 2004). This technique operates at a constant 60–65°C temperature range and utilizes two or three primer sets and a high-displacement activity polymerase to amplify target sequences (Nagamine et al., 2001). This cyclic amplification process generates loop structures within DNA molecules (Nagamine et al., 2002), detectable through visual methods such as turbidity, fluorescence, or color change (Mori et al., 2001; Yoda et al., 2007). The LAMP technique relies on meticulously selecting pathogen genes that exhibit specific recognition without any cross-reactivity to other microorganisms (Wong et al., 2018). Through core genome analysis, pivotal shared genes crucial for fundamental cellular functions in both *P. salmonis* genogroups have been identified. The identification and use of these specific genes within each *P. salmonis* genogroup can significantly advance the rapid genotyping of this pathogen using LAMP assay. Within aquaculture, LAMP assays have successfully been developed for numerous fish pathogens, including *Edwardsiella tarda* (Savan et al., 2005), *Edwardsiella ictaluri* (Yeh et al., 2005), *Flavobacterium columnare* (Yeh et al., 2006), Infectious Hematopoietic Necrosis virus (Gunimaladevi et al., 2004), and Koi herpes virus (Gunimaladevi et al., 2005). The LAMP assays, when applied in aquaculture, show great promise as swift and accurate tools for diagnosing and identifying *P. salmonis* genogroups, thereby facilitating the rapid detection of outbreaks, even across geographically distant locations.

Thus, the aim of this study is to develop three rapid and specific genotyping methods using Loop-mediated Isothermal Amplification (LAMP): one for the accurate identification of the species *P. salmonis* by targeting a common gene shared between genogroups (*ton*B-r), and two others to differentiate between *P. salmonis* genogroups LF-89 (Nitronate monooxygenase) and EM-90 (HAD family acid phosphatase) using unique coding sequences specific to each genogroup. These innovative tools are of critical importance to the salmon industry, as they enable rapid on-site diagnosis at aquaculture facilities. The ability to swiftly identify *P. salmonis* species and distinguish between its genogroups is essential for mitigating the pathogen's impact, as *P. salmonis* infections continue to pose a significant threat, resulting in substantial economic losses within the Chilean salmon aquaculture sector.

## 2 Methodology

### 2.1 Bacterial cultures, identification, and genomic DNA extraction of *P. salmonis*

The *P. salmonis* isolates used for the initial validation of the methodology (Psal-000 to Psal-072) are described later. These samples were isolated from the liver, kidney, and spleen of *S. salar* and *O. kisutch*. The isolates were collected between 1989 and 2017 in southern Chile (Los Lagos and Magallanes Region). They were cultured in SHK-1 cell lines for 10 to 15 days. The culture medium was then centrifuged at 1,000 × g for 5 min. The supernatant was discarded, and the pellet was recovered and cultured on Austral TS-HEM agar and in Austral SRS-broth culture medium (Yañez et al., 2012, 2013). The purity of the cultures was verified by Gram staining, Indirect Fluorescent Antibody Test (IFAT) following the manufacturer's instructions for the Salmonid Rickettsial Syndrome (SRS) Immunofluorescence Kit (Ango), and nested PCR (Mauel et al., 1996; Yañez et al., 2013). Genomic DNA from strains Psal-000 to Psal-072 was extracted using the NucleoSpin Tissue Genomic DNA Purification Kit (Macherey-Nagel), according to the manufacturer's recommendations. DNA quality was visually assessed by agarose gel electrophoresis (0.8% w/v in TAE 1X buffer and SybrSafe, Invitrogen). DNA concentration was adjusted to between 10 and 20 ng/μL using a ScanDrop analyzer (Analytik Jena), and samples were stored at 4°C until further PCR amplification.

### 2.2 Bacterial DNA isolation of marine and freshwater fish pathogens

The specificity of the primer set designed for the identification and genotyping of *P. salmonis* using LAMP and qPCR assays was evaluated with genomic DNA from various pathogenic microorganisms. To underscore the importance and necessity of including specific pathogens in our study, several key salmon pathogens were selected based on their pathogenic relevance, environmental distribution (freshwater and marine), and their role in ensuring assay specificity. This selection significantly contributes to the robustness and accuracy of our diagnostic methods. In this study, *Renibacterium salmoninarum, Vibrio ordalii, Flavobacterium psychrophilum, Tenacibaculum maritimum*, and *Aeromonas salmonicida* were included to evaluate the assay's specificity and cross-reactivity, ensuring accurate differentiation of *P. salmonis from* other common salmonid pathogens. The pure cultures of *R. salmoninarum* and *A. salmonicida* were maintained in our laboratory. *R. salmoninarum* strain SF2022 was cultured on KDM2-1.5% w/v NaCl plate agar for 5 days post-inoculation at 15°C (Evelyn et al., 1989; Flores-Martin et al., 2024). The culture's purity was confirmed by Gram staining and IFAT following the manufacturer's instructions for the Bacterial Kidney Disease Immunofluorescence Kit (Ango). *A. salmonicida* strain AY001 was cultured on blood agar and incubated for 24 h at 18°C. The culture's purity was verified by Gram staining, biochemical tests, and DNA sequencing of the 16S rRNA using the primer set 16SF (5′-AGAGTTTGATCCTGGCTCAG-3′) and 16SR (5′-ACGGATACCTTGTTACGAGTT-3′). The PCR reaction was carried out in a MaxyGene II Thermal Cycler (Axygen Scientific) with the following thermocycling protocol: initial denaturation at 95°C for 3 min, followed by 35 cycles of amplification (denaturation at 95°C for 1 min, annealing at 45°C for 1 min, and extension at 72°C for 2 min), and a final extension at 72°C for 10 min. The PCR products were visualized using agarose gel electrophoresis (1.5% w/v in TAE 1X buffer) and SybrSafe following standard procedures. The PCR products were then recovered from agarose gels using the E.Z.N.A Gel Extraction Kit (Omega-Biotek) according to the manufacturer's instructions. DNA was stored at −20°C until further use. DNA sequencing was performed at AUSTRAL-omics, Universidad Austral de Chile (Valdivia, Chile), using the dideoxynucleotide method (Sanger et al., 1977) with the primers 16SF and 16SR. All chromatograms were manually verified to ensure high sequencing quality.

Genomic DNA from the *R. salmoninarum* and *A. salmonicida* strains was extracted using the NucleoSpin Tissue Genomic DNA Purification Kit (Macherey-Nagel) according to the manufacturer's recommendations. DNA quality was visually assessed by agarose gel electrophoresis (0.8% w/v in TAE 1X buffer and SybrSafe, Invitrogen). DNA concentration was adjusted to between 10 and 20 ng/μL using a ScanDrop analyzer (Analytik Jena), and samples were stored at 4°C until further PCR amplification. Additionally, genomic DNA from *V. ordalii* Vo-18-LM, *F. psychrophilum* NCIMB 1947t, and *T. maritimum* CECT 4276, which were previously donated by Ruben Avendaño-Herrera (Universidad Andrés Bello), was also used to evaluate the specificity of the primer set designed for the identification and genotyping of *P. salmonis*.

### 2.3 Identification of nucleotide and amino acid sequence *ton*B-r

The identification of coding sequences was developed using 73 full complete genome of *P. salmonis* retrieved from NCBI database (https://www.ncbi.nlm.nih.gov/) (Supplementary Table 1). Previously characterized and used to identify coding sequence in an article published by Isla et al. (2021). The shared nucleotide sequence between LF-89 and EM-90 was functionally annotated using the EggNOG-mapper version 2.0.1-14-gbf04860, based on EggNOG 4.5 (http://eggnog-mapper.embl.de/) using default parameters and Diamond for orthologous searches (Buchfink et al., 2015; Huerta-Cepas et al., 2017). The exclusive *P. salmonis* coding sequences were identified using remote BLAST (Madden and Camacho, 2008). Later, nucleotide sequence was translated to amino acids sequence using the Transeq tool (Madeira et al., 2022). The amino acids sequence was annotated against NCBI database using BlastP tool (Altschul et al., 1990) with an E-value < 0.05. Tertiary structure prediction was performed with the I-TASSER server (https://zhanglab.ccmb.med.umich.edu/I-TASSER/) (Yang et al., 2015) and compared with the PDB database using the DALI server (Holm et al., 2023). The crystal structures predictions were visualized using PyMOL 2.5 (The PyMOL Molecular Graphics System, Version 2.5, Schrödinger, LLC). Finally, the *ton*B-r coding sequence in *P. salmonis* (Accession Number: WP_016210144.1), was selected because is a common gen of the specie shared in both genogroups genogroups and do not cross-react to other pathogens (Supplementary Table 2).

### 2.4 Identification of nucleotide and amino acid sequence for genotyping

The coding sequences for genotyping, previously reported by Isla et al. (2021) denominated 1755 and 1207. The confirmation that the nucleotide sequence 1755 and 1207 are exclusive of *P. salmonis* genotype LF-89 and EM-90 respectively was achieved using the BlastN tool against the NCBI database (bacteria and eukaryotic organisms), excluding *P. salmonis* strains of this search (Supplementary Table 2). The nucleotide sequences were translated into amino acid sequences with the EMBOSS Transeq tool (Madeira et al., 2022). The annotation of each amino acid sequence was developed with the BlastP tool against the *P. salmonis* database (Altschul et al., 1990). Moreover, the sequence was functionally annotated using EggNOG-mapper version 2.0.1-14-gbf04860, based on EggNOG 4.5 (http://eggnog-mapper.embl.de/) using default parameters and Diamond for orthologous searches (Buchfink et al., 2015; Huerta-Cepas et al., 2017). The isoelectric point and relative molecular weight of each protein were calculated with the ExPASy server (https://web.expasy.org/protparam/).

### 2.5 Primer design and function for specie identification and genotyping of *P. salmonis* via qPCR and LAMP

Initially, primer sets for amplifying the *ton*B-r coding sequence via qPCR were designed using the Primer-BLAST tool (https://www.ncbi.nlm.nih.gov/tools/primer-blast/) (Ye et al., 2012) with default parameters (Table 1). These primer sets were developed based on the *ton*B-r coding sequence (Accession Number: WP_016210144.1) to identify *P. salmonis*. The *ton*B-r gene was selected because it is common to both the LF-89 and EM-90 genogroups, making it an ideal target for species-level identification.

**Table 1:** List of primers used for identifying and genotyping *P. salmonis* isolates.

**qPCR primers**	**Sequence 5^′^ → 3^′^**	
*ton*B-r_(f)	AGGCTCACGTGTAAACTGGA	
*ton*B-r_(r)	AATGCAGCTGCGTTTCTATACC	
1207_(f)	TGACGAAGCGTATTGTTGCG	
1207-(r)	ACGCTATCGCCACATCATCC	
1755_(f)	ACACCTGTAGTTGCTGCTGG	
1755_(r)	GCAGCTTCAATGCCATTAGCC	
**LAMP primers**		**Sequence 5**′ → **3**′
**EM-90 genogroup**	F3	CGGACGAACAGAAATGTACC
	B3	CTTATATACTCTATCTGCATAACCA
	FIP	AGGGCTTATTATAGTCATTCGGTTTAAAATTTAAAAGCTGCAGGGT
	BIP	CAAGCGATAGTTGCCCAAGGTGCTAAATCACTGTATTGGTC
	Loop F	AAATCAAGCTGCTGCCAAGC
**LF-89 genogroup**	F3	TTCGCTAATACAGAGCCTAA
	B3	GTTCTTTATCGTAAATTGCTTGT
	FIP	CGCGGTTGATGTTTTATTGCTAAATTTTGGAATTGGTTTTATCACTTG
	BIP	TTCGGAGATGTAAAACCATTTGCAGTTTGTACTTGAGTGATTAAGGTAA
	Loop F	TCTGGGACTTGCTCTAACTTC
***ton*****B-r** ***P. salmonis*** **species**	F3	TGTACAAGATGAAACTTCAGG
	B3	GCGAACAATGTCAATTTTTTCG
	FIP	CAAGTCAACGCTACGCAGTGCACTGGAGTTTTACCTAATATTTCC
	BIP	CAGGTACTCGTCGATGGTATTCCGTATCGAGTGTGACAGGAA
	Loop B	ACTTGCTCCCTACAGTCAAAC

The genotyping *P. salmonis* via qPCR, primer sets were designed based on unique coding sequences specific to each genogroup. The Nitronate monooxygenase gene (sequence 1755), specific to the LF-89 genogroup, and HAD family acid phosphatase gene (sequence 1207), specific to the EM-90 genogroup, were selected because they are found exclusively within the differential core genome of their respective genogroups.

Subsequently, primer sets for the LAMP assay specific to *P. salmonis* were designed based on the *ton*B-r coding sequence to ensure broad applicability across different *P. salmonis* strains. For genotyping, LAMP primer sets were specifically designed to target the unique genogroup-specific genes: the Nitronate monooxygenase gene (sequence 1755) for the LF-89 genogroup and the HAD family acid phosphatase gene (sequence 1207) for the EM-90 genogroup. These primers were designed using the NEB LAMP Primer Design Tool version 1.4.1 (New England Biolabs) with default parameters to optimize sensitivity and specificity (Table 1). Each LAMP primer set consisted of six oligonucleotides: two outer primers (F3 and B3), two inner primers (FIP and BIP), and loop primers (LoopF or LoopB). These primers were designed to amplify the target sequences under isothermal conditions, with reactions constant conducted at 65°C for 30 min.

### 2.6 Evaluation of primers by qPCR

The primer set for identification and genotyping of *P. salmonis* by qPCR was performed using genomic DNA. The qPCR was conducted using GoTaq qPCR Master Mix (Promega) with the following amplification protocol on the Stratagene Mx3005P thermal cycler: an initial cycle of 95°C for 10 min, followed by 40 cycles of 15 s at 95°C and 15 s at 60°C.

### 2.7 Application of the LAMP assay with isolates in pure culture and field samples

The optimized LAMP reaction for identifying *P. salmonis* and determining its genogroup (LF-89 or EM-90) was initially tested with 19 *P. salmonis* isolates maintained in our laboratory in pure culture, both in broth and on plate agar (Psal-000 to Psal-072). These isolates had been previously characterized by 16S rDNA sequencing, Multilocus Sequence Typing (MLST), Multiplex PCR, and/or Whole Genome Sequencing (Isla et al., 2021). In addition to pure cultures, the LAMP assay was employed to analyze the presence of *P. salmonis* DNA in 30 tissue samples (liver, kidney, and spleen) from fish infected with *P. salmonis*. The reaction was conducted in a 25 μL volume containing 12.5 μL of WarmStart Colorimetric LAMP 2x Master Mix (New England Biolabs), 60 pmol of each internal primer (FIP and BIP), 5.0 pmol of each external primer (F3 and B3), 30 pmol of the Loop F or Loop B primer, 1.0 μL of DNA, and PCR-grade water to a final volume of 25 μL. The mixture was incubated at 65°C in a thermal incubator (Labnet Accublock Digital Dry Baths) for 30 min, followed by heating to 85°C for 2 min to terminate the reaction. A reaction mixture without DNA was included as a negative control. The LAMP product was visually determined by color change, with yellow indicating positive samples and pink indicating negative samples, as per the manufacturer's instructions. To ensure the quality of the genomic DNA extracted from the infected host tissues, amplification of the elf-1α gene of S. salar was performed by qPCR, using the primer set *elf*-1α_f (5′-CCCCTCCAGGACGTTTACAAA-3′) and *elf*-1α_r (5′-CACACGGCCCACAGGTACA-3′) as previously described by Santibañez et al. (2020). The qPCR was conducted using GoTaq qPCR Master Mix (Promega) with the following amplification protocol on the Stratagene Mx3005P thermal cycler: an initial denaturation at 95°C for 10 min, followed by 40 cycles of 15 s at 95°C and 15 s at 60°C.

## 3 Results

### 3.1 Identification of nucleotide and amino acid *ton*B-r sequence of *P. salmonis*

**Figure 1:**
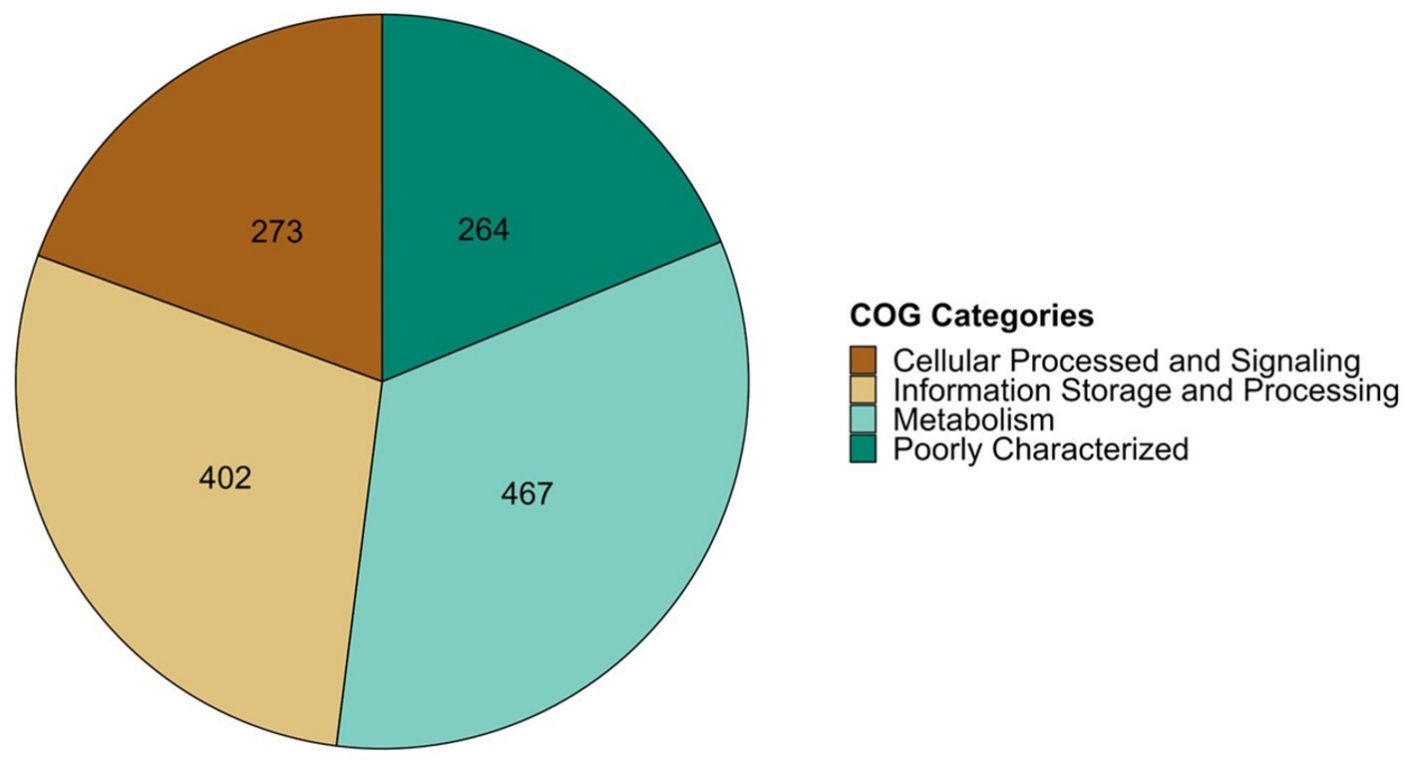
COG classification of share coding sequence of *P. salmonis* group using EggNOG v5.0 tool.

In a previous study, a comprehensive bioinformatic analysis of 73 fully sequenced genomes of *P. salmonis* was conducted, leading to the identification of a shared gene cluster among all *P. salmonis* genomes. This cluster consisted of 1,801 coding sequences that were common to both the LF-89 and EM-90 genogroups, representing the core genome of the specie. The shared sequences were functionally annotated using the COG database with EggNOG-mapper, categorizing them into four main COG categories: Cellular Processes & Signaling, Information Storage & Processing, Metabolism, and Poorly Characterized (E-value < 0.001). The distribution of these categories showed that “Cellular Processes & Signaling” had the fewest annotated sequences (273), while “Metabolism” had the most (467) (Figure 1).

To identify a unique genetic marker for *P. salmonis*, the “Metabolism” COG category was further analyzed using the BlastN tool. The objective was to find nucleotide sequences exclusive to *P. salmonis* that did not match any other microorganisms in the Bacteria kingdom, utilizing the prokaryota database (nt_prok). The analysis revealed 269 nucleotide sequences that were exclusive to *P. salmonis* (E-value < 0.05). These non-matching sequences were subsequently translated into amino acid sequences using the Transeq tool (EMBOSS:6.6.0.0). Among these, the TonB-dependent receptor gene (TonB-r) was selected using the BlastP tool (E-value < 0.05) as a unique genetic marker for the *P. salmonis* species (Accession Number: WP_016210144.1; Supplementary Table 2).

**Figure 2:**
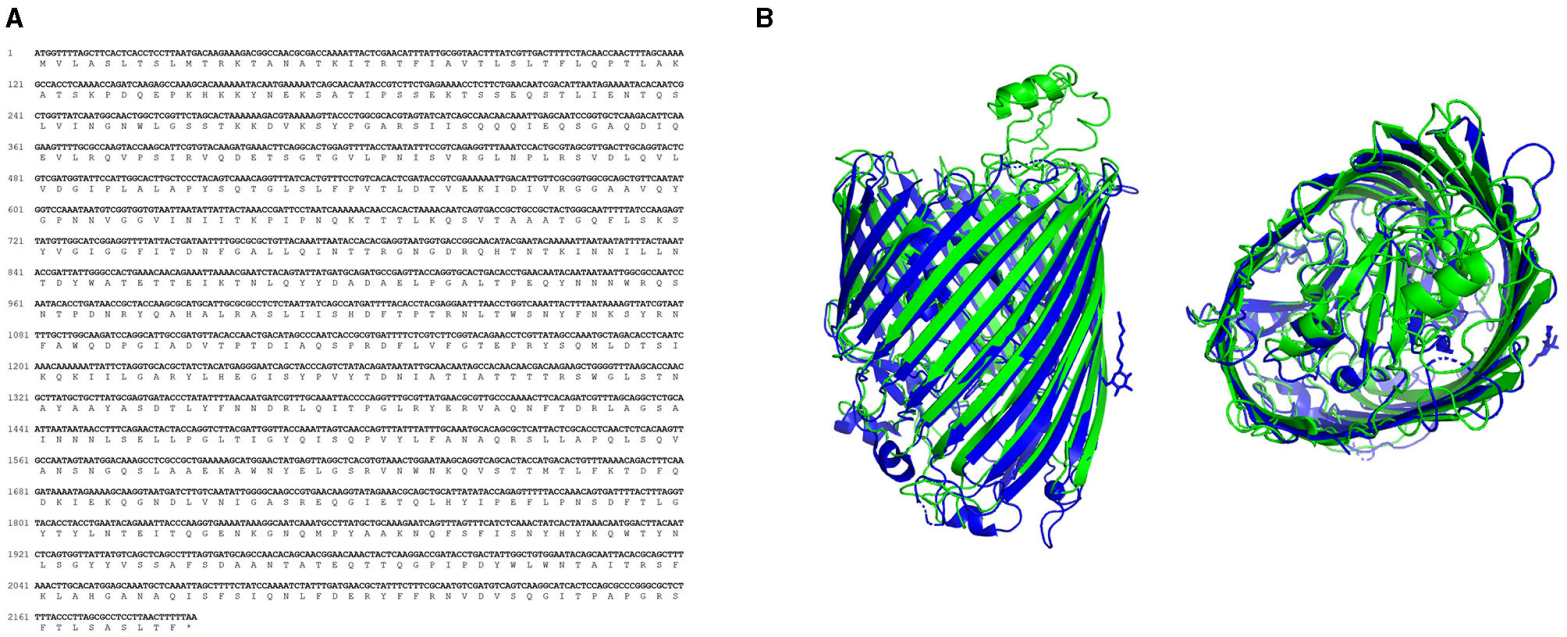
Characterization of TonB-r sequences. **(A)** Nucleotide and amino acid sequence of the predicted sequence. **(B)** Aligned 3D protein model of *P. salmonis* (green) and *E. coli* (blue), obtained using the I-TASSER and DALI servers.

The predicted amino acid sequence of TonB-r (Figure 2A) was compared with sequences available in the NCBI databases. This comparison confirmed 100% identity with 100% coverage to *P. salmonis* sequences (WP_016210144.1) and 61.19% identity with 90% coverage to the *Facilibium subflavum* sequence (WP_119344914.1). Further structural analysis using the I-TASSER server for tertiary structure prediction, followed by comparison with the Protein Data Bank using the DALI server, revealed that the TonB-r protein structure is highly similar to the probable TonB-dependent receptor YNCD of *Escherichia coli* strain BW25113 (Protein Data Bank: 6V81, RMSD 2.96Å) (Figure 2B), with a DALI z-score of 40.4.

**Figure 3:**
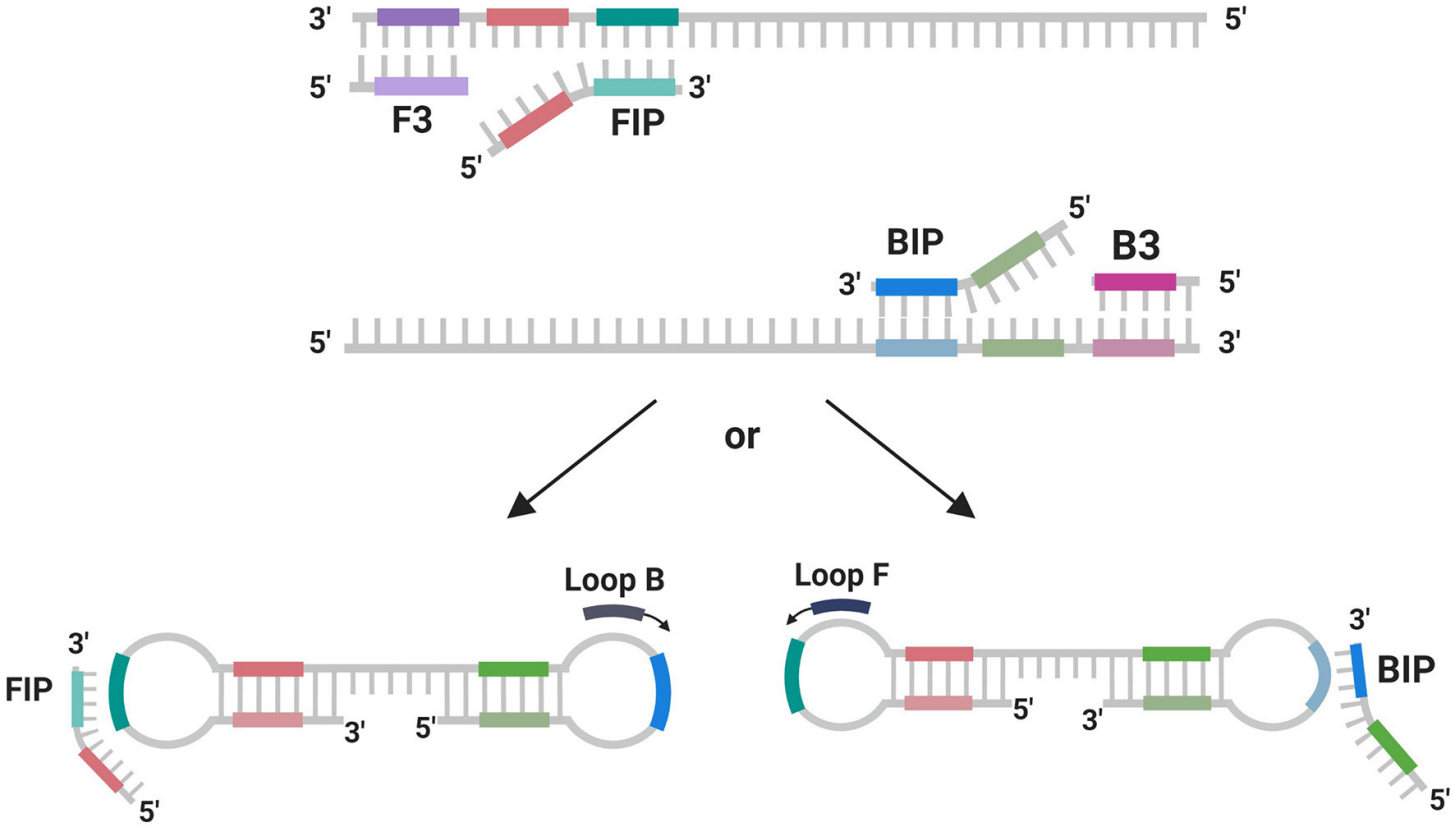
Scheme of primer using in LAMP reaction. Scheme created with BioReder.com.

The TonB-r gene, being highly conserved and specific to the *P. salmonis* species, was selected for the design of primers used in the LAMP assay (Figure 3). This gene's exclusivity, confirmed by the lack of cross-reactivity with any other bacterial sequences in the NCBI database, underscores its suitability as a robust marker for the rapid detection and diagnosis of *P. salmonis* through qPCR and LAMP-specific PCR (Figure 3).

### 3.2 Identification and characterization of unique genotyping coding sequences from core genome

The design of this study was centered on selecting unique genes from the core genome of the LF-89 and EM-90 genogroups of *P. salmonis* that are absent in shared coding sequence between both genogroups. The primary objective was to identify specific genetic markers that are unique to each genogroup, thereby enabling precise genotyping without cross-reactivity (Supplementary Table 2). To achieve this, we conducted a comprehensive analysis using the BlastN tool against the prokaryotic. To achieve this, we conducted a comprehensive analysis using the BlastN tool against the prokaryotic and eukaryotic NCBI databases. This analysis confirmed that the identified coding sequences-−1755 and 1207—are exclusively present in the genomes of their respective genogroups (LF-89 for 1755 and EM-90 for 1207) and have no homology with sequences from other organisms. This specificity was crucial in ensuring that these genes could serve as reliable genotypic markers for differentiating between the two genogroups of *P. salmonis* (Supplementary Table 2).

Furthermore, the coding sequences 1755 and 1207 were translated into their corresponding amino acid sequences and further annotated using BlastP tools and EggNOG-mapper. The 1755 sequence showed 100% amino acid identity with Nitronate monooxygenase (NMO), a unique marker for the LF-89 genogroup (Accession Number: WP_144420689.1), while the 1207 sequence presented 100% amino acid identity with HAD family acid phosphatase (Acid_phosphat_B), specific to the EM-90 genogroup (Accession Number: WP_016210154.1). In addition to confirming their uniqueness to the LF-89 and EM-90 genogroups, further characterization of Nitronate monooxygenase revealed a molecular mass of 20.42 kDa and a theoretical isoelectric point (pI) of 5.43. Similarly, the HAD family acid phosphatase was characterized with a molecular mass of 26.08 kDa and a theoretical isoelectric point of 7.81. Importantly, these target genes did not show any cross-reactivity with sequences from any other bacterial species in the NCBI database, further reinforcing their specificity.

This strategic selection and characterization of unique coding sequences ensure that the identified genetic markers are not only specific to *P. salmonis* but are also exclusive to one of the two genogroups, LF-89 or EM-90. These findings are critical for the development of highly specific and reliable qPCR and LAMP assays that can accurately identify and differentiate the genogroups of *P. salmonis*, ensuring no cross-reactivity with other bacterial species.

### 3.3 Specificity of primer sets for identification and genotyping by qPCR and LAMP assay

The primer sets designed for the identification of *P. salmonis* via qPCR were developed based on the coding sequence of the *ton*B-r gene (Accession Number: WP_016210144.1), which is conserved across both LF-89 and EM-90 genogroups of *P. salmonis*. Additionally, primer sets for genotyping were developed targeting unique genes: Nitronate monooxygenase (sequence 1755) specific to the LF-89 genogroup and HAD family acid phosphatase (sequence 1207) specific to the EM-90 genogroup. The initial standardization of these primers used in the qPCR assay showed a unique amplification product with melting temperature of 79.8°C for *ton*B-r amplicon, 78.5°C for Nitronate monooxygenase (sequence 1755) amplicon and 80.0°C for HAD family acid phosphatase (sequence 1207) amplicon. The efficiency of the primer sets was consistent across multiple experiments, further validating their reliability (data not shown). A comprehensive list of the primers used is presented in Table 1.

Subsequently, these coding sequences were used to design primer sets for the LAMP assay, utilizing the NEB LAMP Primer Design Tool. The results of the LAMP assay for the identification primers targeting *tonB-r* showed a positive reaction with the genomic DNA from both genogroups of *P. salmonis*, confirming the presence of the species across these genogroups. Furthermore, the genotyping-specific LAMP primers targeting Nitronate monooxygenase and HAD family acid phosphatase also showed positive reactions with the DNA from the respective genogroups, with no observed cross-reactivity between the LF-89 and EM-90 genogroups. In this study, LAMP primers were designed based on a single gene sequence from *P. salmonis*, specifically targeting the 3 gene for species and genotyping identification. The design of the LAMP primers involved creating five specific primers—two outer primers (F3 and B3), two inner primers (FIP and BIP), and loop primers (Loop F or Loop B)—which facilitated the amplification of DNA under isothermal conditions. These primers were strategically designed to form loop structures during the amplification process, enhancing the efficiency and specificity of DNA replication, ultimately enabling the rapid and accurate detection of *P. salmonis* in field samples (Figure 3).

The successful amplification and specific reactions in both the qPCR and LAMP assays demonstrate that these primer sets are highly specific and efficient tools for the accurate identification of *P. salmonis* species and the precise genotyping of the LF-89 and EM-90 genogroups. The LAMP assay offers a rapid and reliable alternative to qPCR, with the added benefit of being applicable in less resource-intensive settings. The configuration of the LAMP primer sets is illustrated in the Figure 3.

### 3.4 Specificity of *ton*B-r primer set for identification of *P. salmonis* species

**Figure 4:**
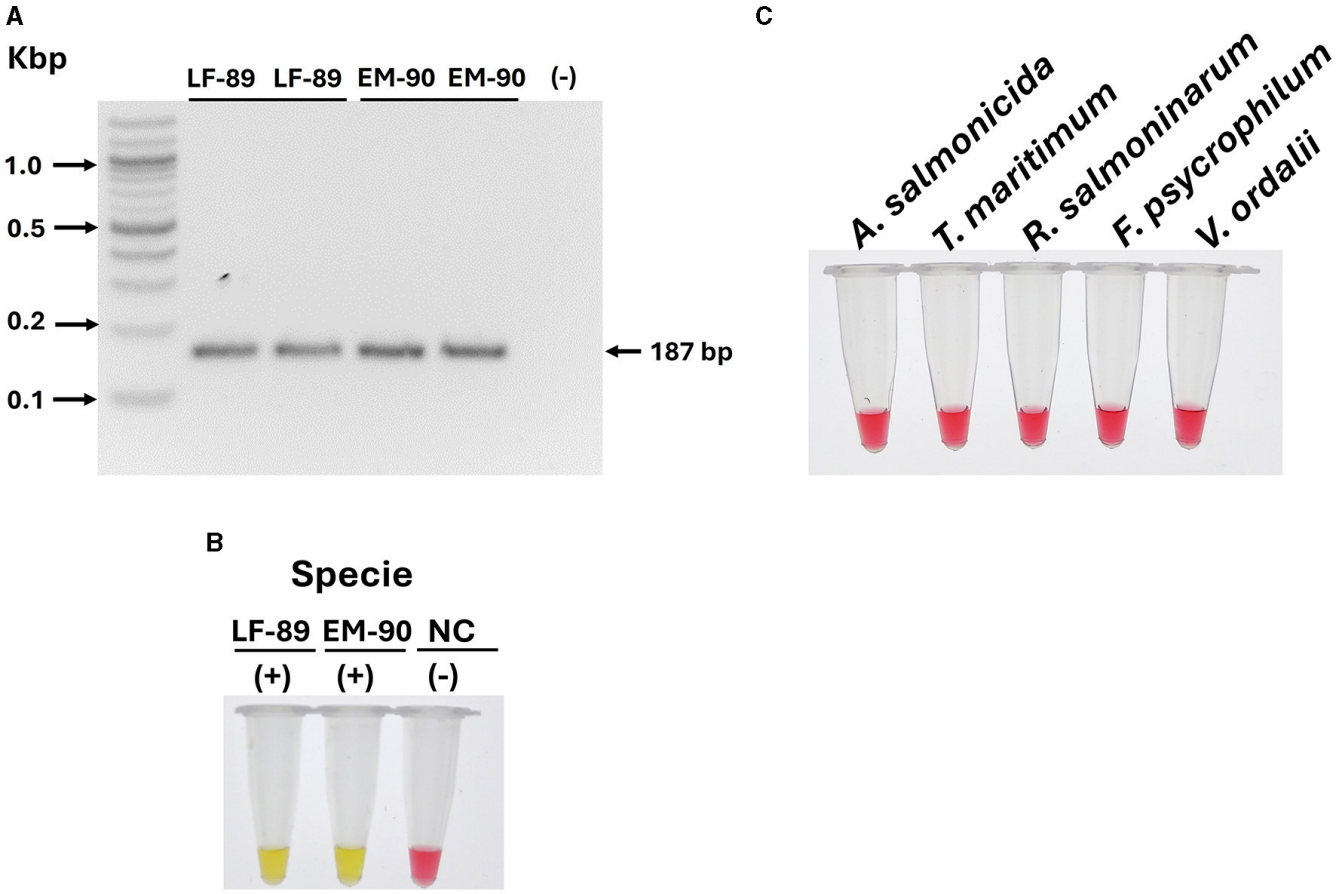
Evaluation of *ton*B-r and primer specificity for the identification of *P. salmonis* species. **(A)** Identification of P. salmonis through conventional PCR. Electrophoresis was performed in a 1.5% w·v^−1^ agarose gel stained with SyberSafe 1x and run in TAE 1x buffer. The gel shows a band at 187 bp, indicating successful amplification in strains LF-89 and EM-90. **(B)** Primer specificity in colorimetric LAMP using genomic DNA from *P. salmonis* LF-89 and EM-90 (positive control) and a negative control (NC). A positive reaction is indicated by a yellow color. **(C)** Specificity of the LAMP assay with genomic DNA from other fish pathogens. A negative reaction is indicated by a pink color.

To assess the specificity of the *ton*B-r primer set for the identification of *P. salmonis* species, we first evaluated the presence of *ton*B-r coding sequences in the genomic DNA of each genogroup (LF-89 and EM-90). Conventional PCR was conducted using a primer set designed specifically for *ton*B-r amplification (Table 1). The PCR results revealed a unique product of 187 bp when using genomic DNA from both genogroups, confirming the presence of the *ton*B-r gene in each (Figure 4A).

Following this, the specificity of the LAMP primer sets designed for *ton*B-r was evaluated using a colorimetric reaction, with yellow indicating a positive reaction and pink indicating a negative reaction. The LAMP assay results demonstrated positive reactions for the identification of *P. salmonis* in both the LF-89 and EM-90 genogroups, with clear yellow color development (Figure 4B).

To further validate the specificity of the *ton*B-r LAMP primer set, we tested the assay using genomic DNA from a variety of other salmonid pathogens, including *A. salmonicida, T. maritimum, R. salmoninarum, F. psychrophilum*, and *V. ordalii*. In all cases, the LAMP assay showed negative reactions (pink color), indicating no cross-reaction with these non-*P. salmonis* species (Figure 4C).

These results confirm that the *ton*B-r primer sets are highly specific for *P. salmonis* species, accurately identifying the pathogen across both LF-89 and EM-90 genogroups without cross-reactivity with other salmonid pathogens. The specificity of the LAMP assay was found to be comparable to that of qPCR, underscoring its reliability as a precise diagnostic tool for the identification of *P. salmonis* species in various applications.

### 3.5 Specificity of primer sets for genotyping LF-89 and EM-90 of *P. salmonis* genogroups

The specificity of the primer sets designed for genotyping the LF-89 and EM-90 genogroups of *P. salmonis* was thoroughly evaluated. Previously, BlastN analysis of the nucleotide sequences for Nitronate monooxygenase (WP_144420689.1) and HAD family acid phosphatase (WP_016210154.1) against the prokaryotic database (nt_prok) confirmed that each gene is uniquely associated with the LF-89 and EM-90 genogroups, respectively. Importantly, these sequences exhibited no significant matches with any other microorganisms (E-value < 0.05).

To validate these findings, a similar analysis was conducted using the updated information available in the NCBI database as of December 2023. The results corroborated the previous conclusions, reaffirming the uniqueness of these genetic markers to their respective genogroups. These specific coding sequences were subsequently utilized to develop primer sets for the LAMP assay (Table 1).

**Figure 5:**
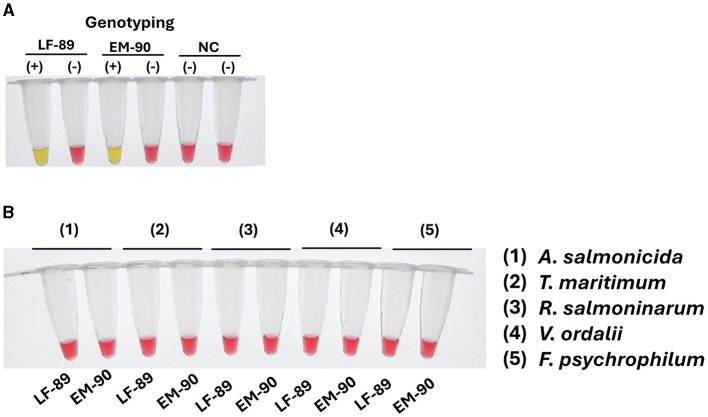
Evaluation of sequence 1207 (Nitronate monooxygenase) and sequence 1755 (Acid_phosphat_B) coding sequence and primer specificity for genotyping *P. salmonis*. **(A)** Primer specificity in colorimetric LAMP using genomic DNA from *P. salmonis* of LF-89 and EM-90 genogroups, and a negative control (NC). A positive reaction is indicated by a yellow color, while a negative reaction is indicated by a pink color. **(B)** Specificity of the LAMP assay with genomic DNA from other fish pathogens (A. *salmonicida, T. maritimum, R. salmoninarum, V. ordalii*, and *F. psychrophilum*). A negative reaction is indicated by a pink color.

The LAMP assay was applied to genomic DNA from both genogroups, resulting in a positive reaction (yellow color) specific to the respective genogroup's DNA, with no cross-reaction observed between the LF-89 and EM-90 genogroups (Figure 5A). Additionally, the specificity of the primer sets was further evaluated using genomic DNA from a range of other relevant fish pathogens, including *A. salmonicida, T. maritimum, R. salmoninarum, F. psychrophilum*, and *V. ordalii*. All these non-*P. salmonis* samples showed a negative reaction (pink color) in the LAMP assay (Figure 5B).

These results confirm that the primer sets for the LAMP assays are highly specific, accurately identifying and genotyping the LF-89 and EM-90 genogroups of *P. salmonis* without cross-reactivity. The specificity of these primers was comparable to that of qPCR, reinforcing the reliability of the LAMP assay as a precise tool for the identification and genotyping of *P. salmonis* genogroups in diagnostic applications.

### 3.6 Sensitivity of *ton*B-r primers for identification of *P. salmonis* species

**Figure 6:**
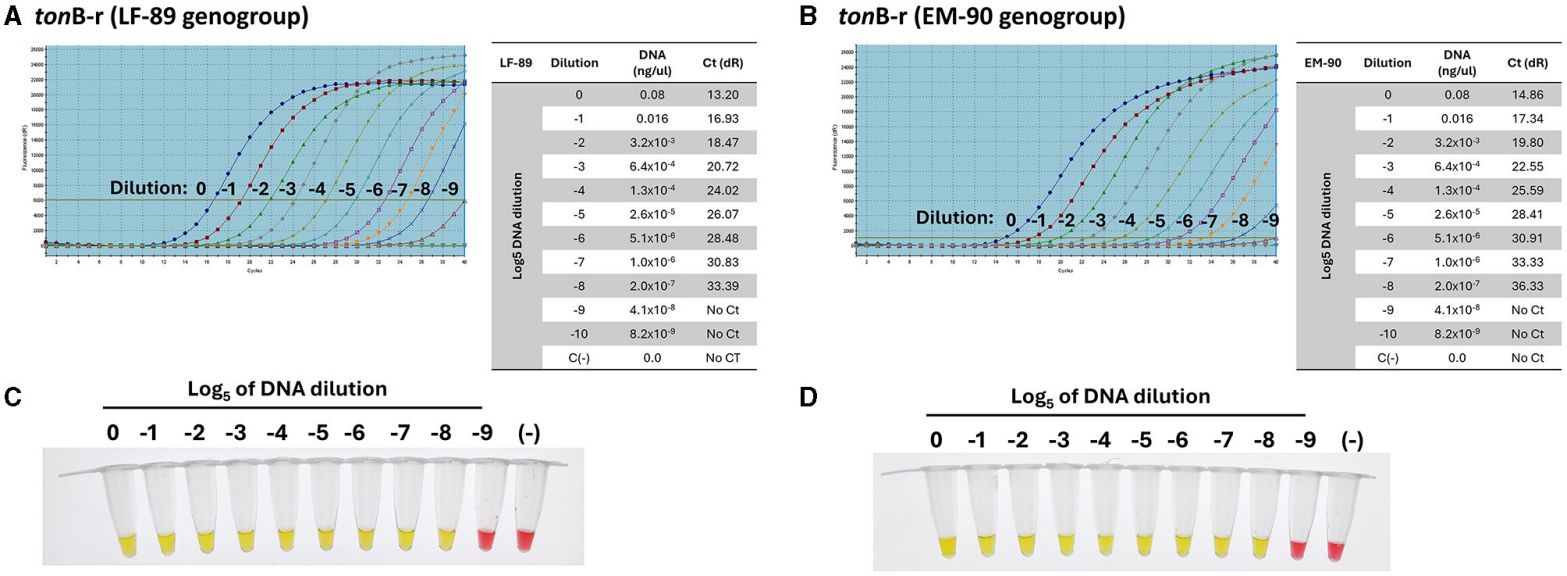
Sensitivity of primers for the detection of *P. salmonis* species by qPCR. **(A, B)** Amplification curves and Ct values of the *ton*B-r sequence using Log_5_ dilutions (0 to −9) of genomic DNA from the LF-89 and EM-90 genogroups, respectively. **(C, D)** Colorimetric LAMP results for Log_5_ dilutions (0 to −9) of genomic DNA from *P. salmonis* LF-89 and EM-90, respectively. Positive reactions are indicated by a yellow color; negative reactions and the negative control (C-) are indicated by a pink color.

The sensitivity of the LAMP assays was directly compared to the established qPCR method, both targeting the *ton*B-r gene (Accession Number: WP_016210144.1), which is common to the LF-89 and EM-90 genogroups of *P. salmonis*. The sensitivity of the primer set designed to amplify the *ton*B-r gene was evaluated using serial dilutions of genomic DNA (Log_5_). Both quantitative PCR (qPCR) and Loop-mediated Isothermal Amplification (LAMP) assays were performed on samples from each *P. salmonis* genogroup (LF-89 and EM-90). The qPCR analysis, using primers specific for the *tonB-r* gene, successfully achieved amplification up to a dilution of (-8) for both genogroups, with Ct values of 33.39 and 36.33 for the LF-89 (Figure 6A) and EM-90 (Figure 6B) genogroups, respectively.

Subsequently, the same range of genomic DNA dilutions was used to assess the sensitivity of the LAMP assay for genotyping. The LAMP results indicated a positive amplification up to a dilution of (−8) for both the LF-89 (Figure 6C) and EM-90 (Figure 6D) isolates. The results demonstrated that the LAMP assays exhibited sensitivity comparable to qPCR, with both methods achieving similar lower detection limits. These findings demonstrate that the LAMP assay, like qPCR, is highly sensitive and capable of detecting low concentrations of *P. salmonis* DNA across both genogroups for identification of *P. salmonis* species. Moreover, the LAMP assay provided the added advantage of faster amplification times under isothermal conditions, making it a practical alternative to qPCR for routine diagnostics.

### 3.7 Sensitivity and specificity of primers for genotyping LF-89 and EM-90 *P. salmonis* genogroup isolates

**Figure 7:**
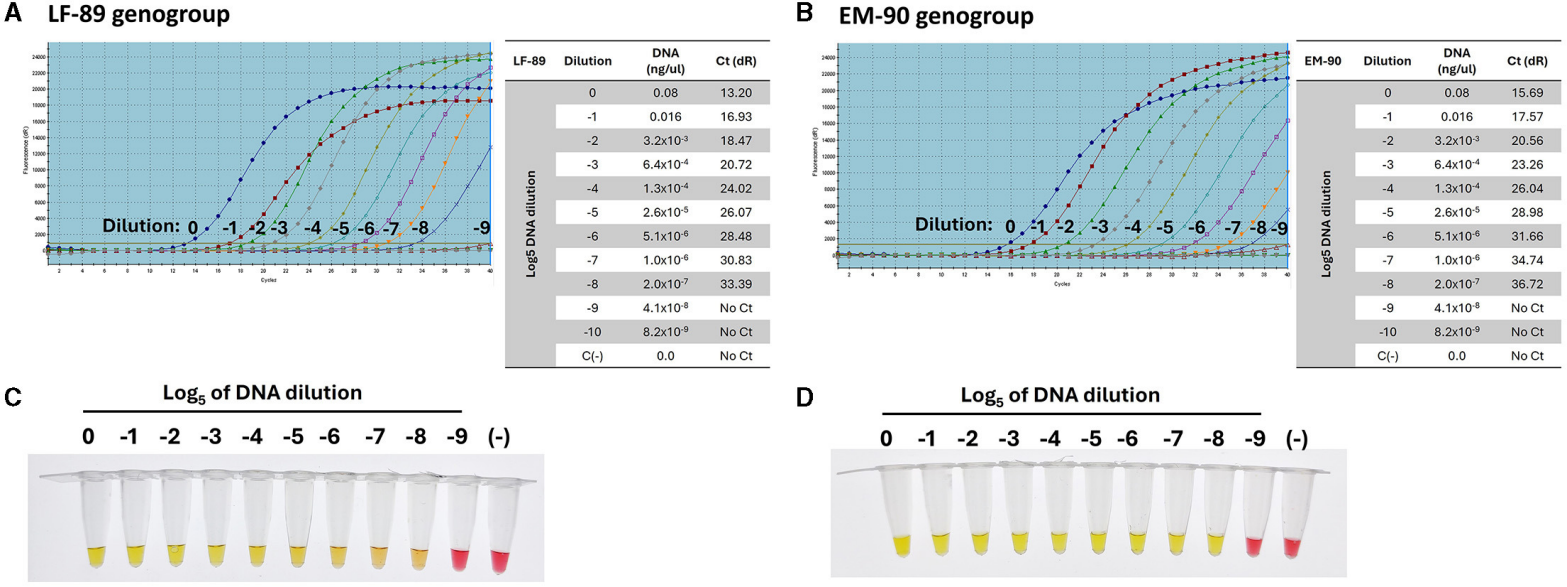
Sensitivity of primers for the detection of *P. salmonis* genogroups (LF-89 and EM-90) by qPCR **(A)** Amplification curve and Ct value of sequence 1755 (Acid_phosphat_B) using Log_5_ dilutions (0 to −9) of the genomic DNA from LF-89 genogroup. **(B)** Amplification curve and Ct value of sequence 1207 (Nitronate monooxygenase) using Log_5_ dilutions (0 to −9) of the genomic DNA from EM-90 genogroup. **(C, D)** Colorimetric LAMP using Log_5_ dilutions (0 to −9) of genomic DNA from *P. salmonis* LF-89 and EM-90, respectively. Positive reactions are indicated by a yellow color, while negative reactions and the negative control (C-) are indicated by a pink color.

The sensitivity and specificity of primer sets for genotyping *P. salmonis* were evaluated using serial dilutions of genomic DNA (Log_5_) from each genogroup. The primers were designed to target Nitronate monooxygenase, a gene unique to the LF-89 genogroup, and HAD family acid phosphatase, a gene unique to the EM-90 genogroup. Using qPCR, the primers for Nitronate monooxygenase demonstrated amplification up to a dilution of (−8) with genomic DNA from the LF-89 strain, yielding a Ct value of 33.39 (Figure 7A). Similarly, the primers for HAD family acid phosphatase amplified DNA from the EM-90 strain up to a dilution of (−8), with a Ct value of 36.72 (Figure 7B). These results confirm the high sensitivity of the qPCR assay for detecting low concentrations of DNA from both genogroups.

Subsequently, the same range of genomic DNA dilutions was used to assess the sensitivity of the LAMP assays for genotyping. The LAMP assays showed positive amplification up to a dilution of (−8) for both the LF-89 isolate (Figure 7C) and the EM-90 isolate (Figure 7D). These findings demonstrate that the LAMP assays exhibit sensitivity comparable to that of qPCR, achieving similar lower detection limits for both genogroups. Moreover, the LAMP assays displayed high specificity, with no cross-reactivity observed between the LF-89 and EM-90 genogroups, further confirming their effectiveness as reliable tools for genotyping *P. salmonis*. The consistency in detection across both methods underscores the robustness of the LAMP assays, making them a valuable alternative to qPCR for routine diagnostics, particularly in resource-limited settings where rapid and specific detection is essential.

### 3.8 Standardization of LAMP-PCR assay with tissue samples from salmon infected with *P. salmonis*

The LAMP assays were initially standardized using genomic DNA from *P. salmonis* isolates maintained in pure culture within our laboratory. These isolates, designated Psal-000 to Psal-073, were collected from various hosts, across different years, and localities in southern Chile. These isolates had been previously genotyped using methods such as 16S rDNA sequencing, MLST, whole genome sequencing, and multiplex PCR. The results demonstrated that all DNA samples from the 20 isolated strains tested positive using the LAMP assay with *ton*B-r primer sets, confirming the success of the newly developed LAMP assay for detecting *P. salmonis* species. This species-specific LAMP reaction was designated as LAMP-PsalSp (LAMP-*P. salmonis* Species) (Table 2), underscoring its efficacy as a reliable tool for species identification.

**Table 2:** Identification and Genotyping of *P. salmonis* using LAMP assay.

**Isolates descriptions**					**LAMP—** ***P. salmonis***		
**Isolate**	**Host**	**Source**	**Year**	**Genotyping methods**	***ton*** **B-r**	**LF-89**	**EM-90**
Psal-000^*^	*O. kisutch*	Liver	1989	(1), (2), (3), (4)	+	+	
Psal-002^**^	*S. salar*	N/D	1990	(1), (2), (3), (4)	+		+
Psal-006	*S. salar*	Kidney	1997	(1), (2), (3), (4)	+	+	
Psal-008	*O. mykiss*	Spleen	1995	(1), (2), (3) (4)	+	+	
Psal-009	*O. kisutch*	N/D	2000	(1), (2), (3) (4)	+	+	
Psal-010	*O. kisutch*	Kidney	1999	(1), (2), (3) (4)	+		+
Psal-012	*S. salar*	Kidney	ND	(1), (2), (3)	+	+	
Psal-015	*S. salar*	Kidney	2002	(1), (2), (3)	+		+
Psal-017	*S sala*	Kidney/liver	2015	(2), (3)	+		+
Psal-018	*S. salar*	Kidney/Liver	2015	(2), (3) (4)	+	+	
Psal-024	*O. kisutch*	Kidney	2008	(2), (3)	+	+	
Psal-034	*S. salar*	Kidney	2017	(3)	+		+
Psal-035	*S. salar*	Liver	2016	(3)	+		+
Psal-036	*O. kisutch*	External injury	2016	(2), (3)	+	+	
Psal-038	*S. salar*	Liver	2016	(2), (3)	+	+	
Psal-061	*S. salar*	Spleen	2017	(3)	+		+
Psal-069	*S. salar*	Kidney/Liver	2009	(2), (3), (4)	+		+
Psal-071	*S. salar*	Kidney	2017	(1), (2), (3), (4)	+		+
Psal-073	*S. salar*	Kidney	2017	(1), (2), (4)	+	+	
Field_01	*S. salar*	Liver	2022	(3)	+	+	
Field_02	*S. salar*	Liver	2022	(3)	+	+	
Field_03	*O. mykiss*	N/D	2022	(3)	+	+	
Field_04	*S. salar*	Liver	2022	(3)	+	+	
Field_05	*S. salar*	Kidney/Liver	2022	(3)	+		+
Field_06	*S. salar*	Kidney/Liver	2022	(3)	+		+
Field_07	*S. salar*	Kidney/Liver	2022	(3)	+		+
Field_08	*S. salar*	Kidney/Liver	2022	(3)	+	+	
Field_09	*S. salar*	Kidney/Liver	2022	(3)	+	+	
Field_10	*S. salar*	Kidney/Liver	2022	(3)	+	+	
Field_11	*S. salar*	Kidney/Liver	2022	(3)	+	+	
Field_12	*S. salar*	Kidney/Liver	2023	(3)	+	+	
Field_13	*S. salar*	Kidney/Liver	2023	(3)	+		+
Field_14	*S. salar*	Kidney/Liver	2023	(3)	+	+	
Field_15	*S. salar*	Kidney/Liver	2023	(3)	+	+	
Field_16	*S. salar*	Kidney/Liver	2023	(3)	+	+	
Field_17	*S. salar*	Kidney/Liver	2023	(3)	+	+	
Field_18	*S. salar*	Kidney/Liver	2023	(3)	+	+	
Field_19	*S. salar*	Kidney/Liver	2023	(3)	+	+	
Field_20	*S. salar*	Kidney/Liver	2023	(3)	+	+	
Field_21	*S. salar*	Kidney/Liver/Spleen	2024	(3)	+	+	
Field_22	*S. salar*	Kidney/Liver	2024	(3)	+	+	
Field_23	*S. salar*	Kidney/Liver	2024	(3)	+	+	
Field_24	*S. salar*	Kidney/Liver/Spleen	2024	(3)	+	+	
Field_25	*S. salar*	Kidney/Liver	2024	(3)	+	+	
Field_26	*S. salar*	Kidney/Liver/Heart	2024	(3)	+	+	
Field_27	*S. salar*	Kidney/Liver	2024	(3)	+	+	
Field_28	*S. salar*	Kidney/Liver	2024	(3)	+	+	
Field_29	*S. salar*	Kidney/Liver/Spleen	2024	(3)	+	+	
Field_30	*S. salar*	Kidney/Liver/Spleen	2024	(3)	+	+	

Isolates obtained from different hosts, source, and years in Chile. Alternative genotyping method used: (1) 16S rDNA sequencing, (2) Multilocus Sequence Tying, (3) Multiplex PCR, (4) Whole genome sequencing. N/D Non data. ^*^LF-89 (ATCC VR-1361) and ^**^EM-90 strain.

Later, the Genotyping-LAMP assays developed with LF-89 (WP_144420689.1) and EM-90 (WP_016210154.1) genes showed that 10 isolates corresponded to the LF-89 genogroup and 9 isolates corresponded to the EM-90 genogroup (Table 2). The results of LAMP-PsalSp and Genotyping-LAMP with isolates maintained in pure culture were consistent with previously published results for these samples.

**Figure 8:**
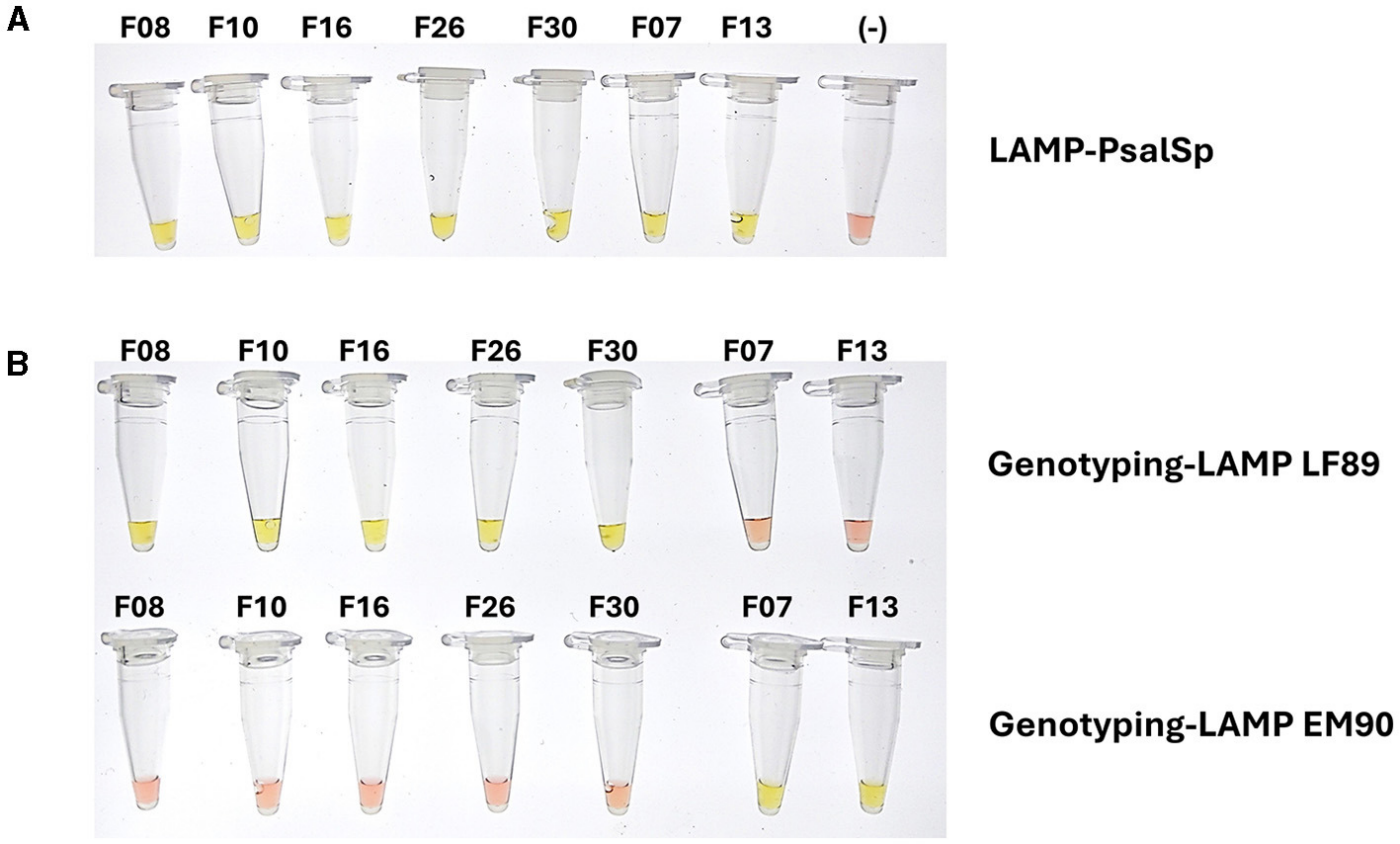
Positive reactions of genomic DNA samples from field isolates of *S. salar* infected with *P. salmonis* using **(A)** LAMP-PsalSp and **(B)** Genotyping-LAMP assays. The field samples were collected from liver, kidney, spleen, heart tissues between 2022 and 2024, confirming the presence of *P. salmonis* and LF-89 and EM-90 genogroups.

Subsequently, DNA or cDNA extractions from 30 tissue samples from salmon infected with *P. salmonis* from the field aquaculture. The 30 tissue samples were PCR positively identified with *P. salmonis* infection, were re-evaluated using the LAMP-PsalSp and Genotyping-LAMP assays. These field samples were designated Field_01 to Field_30. The genomic DNA or cDNA samples were obtained from the liver, kidney, spleen, and heart of *S. salar* between 2022 and 2024. The quality of DNA extraction or cDNA preparation was evaluated through the amplification of *elf*-1α of the host using qPCR. The samples showed positive Ct values between 22 and 25 (Supplementary Figure 1). Subsequently, the 30 field samples that were positive for *P. salmonis* in fish were re-tested using qPCR, targeting the *ton*B-r gene as a specific marker. The results confirmed that the *S. salar* field samples were indeed positive for *P. salmonis* infection. Additionally, the results indicated that all 30 samples showed positive reactions with LAMP-PsalSp. Finally, genotyping the DNA of the field samples with the new Genotyping-LAMP assays revealed that 26 samples showed positive reactions for the LF-89 genogroup, while 4 samples showed positive reactions for the EM-90 genogroup (Table 2). A representative set of field samples showing positive reactions with both LAMP-PsalSp and Genotyping-LAMP is presented in Figure 8.

## 4 Discussion

This study successfully developed and validated Loop-mediated Isothermal Amplification (LAMP) assays specifically designed for the rapid and accurate identification and genotyping of *P. salmonis*, a major pathogen in salmonid aquaculture. The LAMP-PsalSp assay, targeting the *ton*B-r gene, demonstrated high sensitivity and specificity across various *P. salmonis* isolates, while the Genotyping-LAMP assays effectively differentiated between the LF-89 and EM-90 genogroups using unique genetic markers, namely Nitronate monooxygenase and HAD family acid phosphatase, respectively. These findings underscore the potential of LAMP as a reliable and efficient diagnostic tool, offering significant advantages over traditional methods in both specificity and operational simplicity. In the broader context of microbiology and aquaculture, the development of these assays represents a crucial advancement for disease management, enabling timely interventions that are critical for reducing economic losses in the aquaculture industry (Notomi et al., 2000; Abdelsalam et al., 2022).

Aquaculture continues to grapple with persistent challenges posed by infectious diseases, notably Piscirickettsiosis caused by *P. salmonis*, which remains a significant threat to the global salmonid industry (Maisey et al., 2017; Rozas and Enriquez, 2014; Figueroa et al., 2019). Traditional efforts to address these challenges have largely relied on standard species-specific RT-PCR conducted in formal laboratory settings, which are essential for controlling the quality of diagnostic procedures within the salmon market (Karatas et al., 2008). In this context, a standardized gold-standard PCR method is critical for ensuring consistent and reliable diagnostic results across different laboratories, particularly when compared with government reference laboratories. However, such standardization is currently lacking for *P. salmonis* and many other pathogens in Chile, highlighting an urgent need for new, accessible, and standardized diagnostic methods recognized by authorities. These methods would enable swift and accurate pathogen identification and genotyping, particularly for *P. salmonis* and other salmon pathogens, and could be adopted as a reference gold standard for PCR diagnosis, ensuring consistency across various laboratories (Isla et al., 2021).

Recent advancements have led to the development of LAMP assays, which offer rapid on-site diagnostic capabilities. These innovative tools are particularly crucial for the salmon industry, as they enable quick species identification and differentiation of *P. salmonis* genogroups (Parida et al., 2008; Biswas and Sakai, 2014). Such advancements are essential to mitigate the impact of *P. salmonis* infections, which continue to pose a major threat and cause substantial economic losses within the aquaculture industry (Ramírez et al., 2015; Lannan and Fryer, 1993).

This study thoroughly explores the development and validation of innovative diagnostic and genotyping techniques using LAMP assays—one tailored for *P. salmonis* species (LAMP-PsalSp) and two for genotyping (Genotyping-LAMP-LF89 and Genotyping-LAMP-EM90 assays). Recognizing the need for a practical, cost-effective, and field-adaptable genotyping tool, our investigation leverages LAMP's specificity and sensitivity to differentiate between the distinctive LF-89 and EM-90 genogroups (Saavedra et al., 2017; Nourdin-Galindo et al., 2017). This differentiation is crucial for a better understanding of the bacterium's epidemiology and pathogenicity (Rozas-Serri et al., 2017). The manuscript details the methodology, validation process, and implications of this groundbreaking genotyping approach, marking a significant advancement in disease surveillance and management within the aquaculture sector.

While PCR and qPCR are established methods for genotyping *P. salmonis* genogroups, their reliance on sophisticated lab equipment and potential primer interference limit their practicality in field settings (Isla et al., 2021; Soroka et al., 2021; Kreitmann et al., 2023; Ador et al., 2021). Loop-mediated isothermal amplification (LAMP), a recent isothermal nucleic acid amplification technique, offers a swift, cost-effective, and precise alternative for various applications, including pathogen identification and genetic disorder detection (Notomi et al., 2000). Unlike PCR, LAMP operates at a constant temperature (60 to 65°C), employing two or three sets of primers and a polymerase with robust strand displacement activity (Nagamine et al., 2001). Recognizing the importance of a comprehensive comparison, we have expanded the discussion to include a detailed quantitative and qualitative analysis of the LAMP method relative to other established techniques such as PCR, qPCR, sequencing, phenotypic identification, and MALDI TOF MS. This comparison highlights the advantages of the LAMP method, particularly in terms of cost-effectiveness, time efficiency, technical expertise required, and applicability in field settings.

The LAMP method offers distinct advantages over traditional diagnostic techniques such as PCR, qPCR, sequencing, phenotypic identification, and MALDI TOF MS. LAMP's constant-temperature operation eliminates the need for expensive thermal cyclers and allows for rapid amplification with results available in under an hour, contrasting with the longer processing times required by PCR and sequencing (Notomi et al., 2000; Parida et al., 2008; Abdelsalam et al., 2022). Unlike sequencing and MALDI TOF MS, which demand specialized, costly equipment and highly trained personnel, LAMP can be performed with minimal technical expertise and basic laboratory infrastructure, making it both cost-effective and accessible for field applications (Mori et al., 2001; Croxatto et al., 2012; Patel, 2015; Soroka et al., 2021). Additionally, LAMP's visual detection via color change offers straightforward interpretation of results, contrasting with the more complex data analysis needed for sequencing and MALDI TOF MS, further reducing costs associated with technical training and equipment (Nagamine et al., 2002; Yeh et al., 2006). LAMP's lower reagent and equipment costs make it an affordable option for routine diagnostics, especially in resource-limited settings (Notomi et al., 2000). Moreover, LAMP's rapid processing time—delivering results in under an hour—compares favorably against the several hours required for PCR and qPCR, and the days needed for sequencing (Parida et al., 2008). LAMP can be performed with minimal technical expertise and without the need for complex thermal cyclers or sequencing platforms, making it accessible and practical for field settings (Nagamine et al., 2002).

The LAMP assay was designed to target a coding genetic sequence shared between the LF-89 and EM-90 isolates, enabling the specific diagnosis of the *P. salmonis* species. The gene consistently present in all genomes of *P. salmonis* without mutations was the receptor of TonB. Part of its nucleotide sequence is unique to *P. salmonis* and not found in other bacteria present in the NCBI database. The novel LAMP-PsalSp assay uses new *ton*B-r primers targeting the TonB-dependent receptor (TonB-r). This protein, known for facilitating siderophore transport across membranes, is present in both genogroups and represents a species-specific gene (Nourdin-Galindo et al., 2017; Noinaj et al., 2010; Fujita et al., 2019; Briat, 1992; Collins, 2003).

The *ton*B-r gene forms the foundation for developing the LAMP-PsalSp assay, designed for the rapid and comprehensive diagnosis of the entire *P. salmonis* species. This assay has demonstrated high sensitivity and specificity by targeting genomic DNA unique to *P. salmonis*, effectively avoiding amplification of DNA from other fish pathogens such as *A. salmonicida, T. maritimum, R. salmoninarum, F. psychrophilum*, and V. *ordalii*. The absence of cross-reactivity with these non-*P. salmonis* pathogens underscores the LAMP assay's precision as a diagnostic tool. This high specificity is crucial in environments where multiple pathogens coexist, as cross-reactivity could compromise diagnostic accuracy. Similar studies have highlighted the need for assays with stringent specificity to prevent false positives, which are critical for reliable disease management in aquaculture (Yu et al., 2013; Biswas and Sakai, 2014). This comprehensive approach validates the LAMP assay as a swift and accurate diagnostic method that can be readily employed in the field, facilitating timely intervention and effective management of Piscirickettsiosis, ultimately reducing economic losses in the salmon farming industry. The results of our study underscore the effectiveness and reliability of the LAMP assays for detecting and genotyping *P. salmonis* in both cultured isolates and field samples. The LAMP-PsalSp assay successfully identified *P. salmonis* in 30 field samples, confirming the presence of the pathogen through the specific targeting of the *ton*B-r gene. This demonstrates the assay's robustness in field conditions, aligning with previous validations using RT-qPCR. Genotyping with the newly developed LAMP assays revealed distinct genogroups within the samples, with the majority aligning with the LF-89 genogroup and a smaller subset corresponding to the EM-90 genogroup. This differentiation is crucial for epidemiological tracking and understanding the distribution of *P. salmonis* genotypes in southern Chile. The validation of these assays against a well-characterized collection of *P. salmonis* isolates, previously genotyped through methods such as 16S rDNA sequencing, MLST, multiplex PCR and whole genome sequencing, further solidifies their accuracy and utility. The consistent Ct values obtained from qPCR amplification of *elf*-1α of the host using genomic DNA or cDNA in field samples reinforce the quality and integrity of the DNA used in our analyses. Overall, the LAMP assays, particularly LAMP-PsalSp and the genotyping assays, provide reliable, rapid, and specific tools for the identification and differentiation of *P. salmonis* strains in diverse sample types, which is essential for effective disease management and control in aquaculture.

Moreover, we developed and standardized the first qPCR assay for *P. salmonis*, targeting *ton*B-r gene a uniquely characterized gene shared by both genogroups of *P. salmonis*. This gene is distinct, showing minimal identity to other bacteria, making it a unique marker in the bacterial world that exclusively recognizes the *P. salmonis* species. This remarkable discovery led to the establishment of this qPCR assay as the gold standard for diagnostic laboratories, allowing all labs to utilize the same standardized conditions, rather than relying on varied, homemade primers and protocols to detect this pathogen.

Furthermore, this gene sequence identification strategy serves as the basis for developing genotyping-LAMP assays for *P. salmonis*. These assays utilize distinct coding sequences unique to each genogroup, identified through genomic comparisons that highlight exclusive genes in each group. For instance, Nitronate monooxygenase (WP_144420689.1) characterizes LF-89, while HAD family acid phosphatase (WP_016210154.1) defines EM-90. The genotyping-LAMP assays demonstrated comparable specificity and sensitivity in detecting LF-89 and EM-90 sequences, using both LAMP and qPCR, the gold standard assay. These findings confirm the precision of the primer sets in distinguishing LF-89 and EM-90 genogroups of *P. salmonis*, highlighting their non-reactivity with DNA from other pathogenic fish bacteria (Isla et al., 2021). This reaffirms the high sensitivity and specificity of the genotyping methodology. Additionally, should there be a need for the development of new genotyping PCRs, more unique proteins specific to each genogroup have been identified and can be utilized (Isla et al., 2021). The presence of Nitronate monooxygenase (WP_144420689.1) in *P. salmonis* genogroup LF-89 is essential for the detoxification of nitroalkanes, contributing to the organism's ability to manage oxidative stress and enhance virulence within the host environment. This enzyme catalyzes the oxidation of nitronates to aldehydes or ketones, which is crucial for nitrogen metabolism and the detoxification pathways necessary for survival under stress conditions. The enzyme's role in virulence is supported by similar findings in other pathogens, where nitronate monooxygenases contribute to managing oxidative stress and facilitating survival in hostile environments (Nguyen et al., 2022; Vodovoz and Gadda, 2020). The unique coding sequence of Nitronate monooxygenase makes it an excellent genetic marker for differentiating LF-89 from other genogroups, such as EM-90, enhancing diagnostic precision in tools like LAMP assays.

The absence of Nitronate monooxygenase in the EM-90 genogroup of *P. salmonis* may indicate an adaptation to environments where nitroalkane detoxification is less critical, or where alternative environmental factors have reduced the necessity for this function. This genogroup likely thrives in niches where the substrates of Nitronate monooxygenase are not prevalent, or where other environmental pressures have favored the loss or replacement of this enzyme. Furthermore, EM-90 may have evolved distinct virulence factors or stress response mechanisms to compensate for the absence of Nitronate monooxygenase, providing sufficient protection against oxidative stress or other challenges encountered within the host (Porrini et al., 2021; Riffaud et al., 2023). These adaptive strategies underline the genetic diversity and evolutionary plasticity of *P. salmonis* genogroups, each optimizing its metabolic processes and survival strategies according to the specific environments and hosts it encounters (Rozas-Serri, 2022).

The presence of Acid phosphatase (WP_016210154.1) in *P. salmonis* genogroup EM-90 suggests its crucial role in bacterial survival and pathogenicity. Acid phosphatases are enzymes that play a significant role in bacterial physiology by catalyzing the hydrolysis of phosphate esters, which can be vital for bacterial adaptation to diverse environmental conditions. In pathogenic bacteria, these enzymes are often associated with the ability to survive within host cells by contributing to the detoxification of reactive oxygen species and aiding in the evasion of host immune defenses (Ahmad-Mansour et al., 2022). In the case of *P. salmonis* EM-90, Acid phosphatase might be involved in modulating the intracellular environment to favor bacterial survival and proliferation, particularly under conditions of oxidative stress encountered within the host. This enzymatic activity is crucial for the pathogen's virulence, enabling it to persist in hostile environments where phosphate availability is limited or where the pathogen needs to neutralize the acidic conditions within phagosomes (Dai et al., 2012; Ahmad-Mansour et al., 2022). The absence of Nitronate monooxygenase in EM-90, as discussed earlier, might be compensated by the presence and function of Acid phosphatase, which could provide alternative mechanisms to manage oxidative stress and maintain metabolic homeostasis. This adaptation highlights the genetic diversity and flexibility of *P. salmonis* genogroups, each evolving distinct strategies to optimize their survival and pathogenic potential within specific environmental niches and host interactions (Udaondo et al., 2020).

Moreover, the results presented provide compelling evidence that the *ton*B-r primer sets, along with the LAMP assays designed for the LF-89 and EM-90 genogroups, are highly specific for *P. salmonis*. Our study demonstrated that these primers accurately identify the pathogen across both genogroups without cross-reactivity with other salmonid pathogens. Specifically, when the LAMP assay was applied to genomic DNA from a variety of significant salmonid pathogens, including *A. salmonicida, T. maritimum, R. salmoninarum, F. psychrophilum*, and *V. ordalii*, all tests showed negative reactions (pink color), confirming no cross-reaction with non-*P. salmonis* species. These findings underscore the specificity of the *ton*B-r LAMP primer set and its reliability as a diagnostic tool comparable to qPCR for the identification of *P. salmonis* in various applications.

Furthermore, the positive reactions observed with the genomic DNA from both LF-89 and EM-90 genogroups (yellow color) and the absence of cross-reaction between these genogroups further validate the specificity of the genotyping LAMP assays. The robustness of these assays is particularly significant given that *R. salmoninarum* and *A. salmonicida* are well-established salmonid pathogens, responsible for bacterial kidney disease (BKD) and furunculosis, respectively. Both pathogens have significant impacts on salmonid health, similar to *P. salmonis*, but the absence of cross-reactivity in our assays underscores the precision of the LAMP technique (Zhou et al., 2021). Importantly, *R. salmoninarum* primarily inhabits freshwater environments, while *A. salmonicida* is found in both freshwater and marine environments, analogous to the habitat of *P. salmonis*. Including these pathogens as controls in the specificity testing ensures that the LAMP assays developed for *P. salmonis* are not only effective but also do not mistakenly detect other common salmonid pathogens from diverse aquatic environments (Biswas and Sakai, 2014).

The inclusion of such comprehensive controls is essential for validating the exclusivity of the LAMP assays. The demonstrated lack of detection for these non-*P. salmonis* pathogens further emphasizes the accuracy of the LAMP method, ensuring it is exclusively sensitive and specific for *P. salmonis*. This specificity is reinforced by the *ton*B-r coding sequence uniqueness to *P. salmonis*, as confirmed by BlastN analysis against the NCBI database, which showed no significant similarity to other bacterial species. Moreover, the LAMP assay's ability to rapidly and specifically detect *P. salmonis* without cross-reaction, even in the presence of other closely related pathogens, aligns with findings in recent studies focused on the development of isothermal amplification methods for pathogen detection (Shen et al., 2020).

LAMP assays have been successfully developed for various fish pathogens, such as *E. tarda* (Savan et al., 2005), *E. ictaluri* (Yeh et al., 2005), *F. columnare* (Yeh et al., 2006), infectious hematopoietic necrosis virus (Gunimaladevi et al., 2004), koi herpes virus (Gunimaladevi et al., 2005), and *R. salmoninarum* (Saleh et al., 2008). The LAMP method, utilizing five primers recognizing six DNA sequences, has shown remarkable specificity (Notomi et al., 2000). Its precision and sensitivity were validated by detecting specific genes like P57 in *R. salmoninarum* DNA, demonstrating sensitivity comparable to nested PCR for the same gene (Saleh et al., 2008). Therefore, these reported outcomes align with previous successes in identifying pathogens within aquaculture.

The LAMP assay developed in this study for the detection of *P. salmonis* was rigorously standardized using genomic DNA from well-characterized isolates, showing robust performance in identifying the pathogen across various samples. The assay's specificity was further confirmed by its ability to distinguish *P. salmonis* without cross-reactivity with other significant salmonid pathogens, such as *A. salmonicida, T. maritimum, R. salmoninarum, F. psychrophilum*, and *V. ordalii* (Figueroa et al., 2019). The pH-dependent colorimetric change from pink to yellow provided a clear visual indication of a positive result, making it suitable for field applications (Wong et al., 2018). When applied to field samples, the LAMP-PsalSp assay consistently detected *P. salmonis*, and the genotyping LAMP assays accurately identified the LF-89 and EM-90 genogroups, confirming their utility as reliable diagnostic tools. This study underscores the LAMP assay's advantages, including its rapid detection, simplicity, and ability to function under isothermal conditions, which are particularly advantageous for on-site diagnostics in aquaculture environments (Adams and Thompson, 2011; Biswas and Sakai, 2014; Wong et al., 2018).

## 5 Conclusion

This study successfully developed and validated innovative diagnostic and genotyping techniques using Loop-mediated Isothermal Amplification (LAMP) assays specifically designed for *P. salmonis*. The LAMP-PsalSp assay, which targets the conserved *tonB-r* gene, provides a rapid and highly sensitive method for diagnosing the entire *P. salmonis* species. Its high specificity ensures quick on-site identification, which is crucial for effective management of Piscirickettsiosis in the aquaculture industry. Additionally, the Genotyping-LAMP assays, tailored to differentiate between the distinctive LF-89 and EM-90 genogroups, target unique coding sequences—Nitronate monooxygenase for LF-89 and Acid phosphatase for EM-90. These assays demonstrated specificity and sensitivity comparable to qPCR, confirming their precision in genotyping *P. salmonis*. The validated use of the *tonB-r* and genotyping LAMP assays underscores their value as precise, specific, and reliable tools for diagnosing *P. salmonis* and differentiating between its genogroups, offering clear advantages over traditional methods in terms of speed, specificity, and applicability in diverse environments. The ability to rapidly genotype *P. salmonis* isolates using these assays is pivotal for understanding the bacterium's epidemiology and pathogenicity, thereby enhancing disease management and control strategies within the salmon aquaculture sector.

These advancements mark a significant step forward in the rapid detection, diagnosis, and genotyping of *P. salmonis*, promising to improve disease surveillance and mitigate economic losses in aquaculture.

## Data Availability

The dataset analyzed for this study can be found in the Genbank (https://www.ncbi.nlm.nih.gov/genbank/), and accession numbers can be found in the article and Supplementary material (Supplementary Table 1).
