# Supplementary material for: Advancements in rapid diagnostics and genotyping of Piscirickettsia salmonis using Loop-mediated Isothermal Amplification
Source: Front Microbiol. 2024 Sep 24;15:1392808. doi: 10.3389/fmicb.2024.1392808 (PMC11458457; doi:10.3389/fmicb.2024.1392808)
Supplement: Supplementary file 1 [file Table_1.DOCX]

Supplementary Material

##
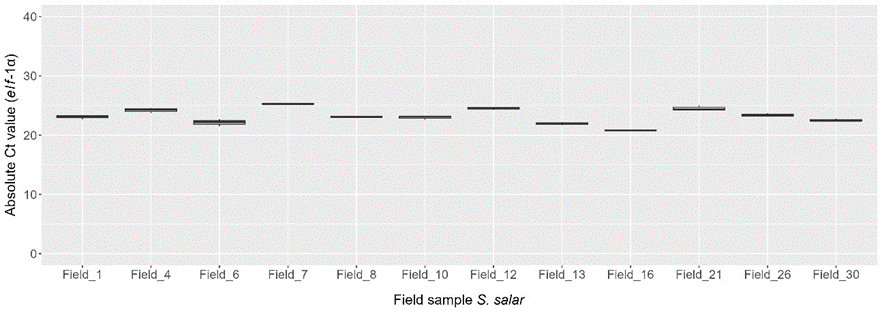
Supplementary Figure

**Supplementary Figure 1.** Ct value of amplification of *elf*-1a using genomic DNA of *S. salar* from field samples infected with *P. salmonis*

## Supplementary Tables

**Supplementary Table 1**. Accession number of 73 fully sequenced genomes of *Piscirickettsia salmonis* used in the identification of shared coding sequence. Genomes available at the NCBI database (Isla et al., 2021).

| **Accession number** | **Strain** | **Length (Mb)** | **Host** |
| --- | --- | --- | --- |
| CP011849.2 | LF-89 ATCC VR-1361 | 3.51 | *Oncorhynchus kisutch* |
| CP039240.1 | MR5 | 4.15 | *Salmo salar* |
| CP039227.1 | SR1 | 4.15 | *Salmo salar* |
| CP039234.1 | BI1 | 4.07 | *Salmo salar* |
| CP038908.1 | Psal-009 | 3.56 | *Oncorhynchus kisutch* |
| CP039219.1 | NVI 5692 | 3.76 | *Salmo salar* |
| CP050938.1 | Ps-2192A | 3.70 | *Salmo salar* |
| CP038927.1 | Psal-013 | 3.49 | *Salmo salar* |
| CP048056.1 | Ps-8942B | 3.71 | *Salmo salar* |
| CP013975.1 | CGR02 | 3.42 | *Salmo salar* |
| CP038904.1 | Psal-008 | 3.45 | *Oncorhynchus mykiss* |
| CP038898.1 | Psal-006b | 3.60 | *Salmo salar* |
| CP039060.1 | Psal-099 | 3.51 | *Oncorhynchus mykiss* |
| CP038918.1 | Psal-010b | 3.51 | *Oncorhynchus kisutch* |
| CP039055.1 | Psal-098 | 3.51 | *Oncorhynchus mykiss* |
| CP039112.1 | Psal-135 | 3.52 | *Oncorhynchus mykiss* |
| CP039087.1 | Psal-111 | 3.51 | *Oncorhynchus kisutch* |
| CP039107.1 | Psal-134 | 3.52 | *Oncorhynchus mykiss* |
| CP039097.1 | Psal-117 | 3.50 | *Oncorhynchus kisutch* |
| CP039171.1 | Psal-138 | 3.51 | *Oncorhynchus mykiss* |
| CP039868.1 | Psal-113 | 3.50 | *Oncorhynchus kisutch* |
| CP039076.1 | Psal-109 | 3.51 | *Oncorhynchus kisutch* |
| CP039176.1 | Psal-139 | 3.51 | *Oncorhynchus mykiss* |
| CP039070.1 | Psal-108 | 3.53 | *Oncorhynchus kisutch* |
| CP039092.1 | Psal-114 | 3.51 | *Oncorhynchus kisutch* |
| CP048052.1 | Ps-11091B | 3.52 | *Oncorhynchus kisutch* |
| CP013786.1 | PM58386B | 3.52 | *Salmo salar* |
| CP039102.1 | Psal-118 | 3.51 | *Oncorhynchus kisutch* |
| CP013806.1 | PM31429B | 3.52 | *Oncorhynchus mykiss* |
| CP013781.1 | PM49811B | 3.52 | *Salmo salar* |
| CP039081.1 | Psal-110 | 3.52 | *Oncorhynchus kisutch* |
| CP039046.1 | Psal-073 | 3.55 | *Salmo salar* |
| CP013811.1 | AY3864B | 3.52 | *Salmo salar* |
| CP013791.1 | AY6297B | 3.52 | *Salmo salar* |
| CP013796.1 | AY6532B | 3.49 | *Salmo salar* |
| CP039050.1 | Psal-081 | 3.51 | *Oncorhynchus mykiss* |
| CP013801.1 | PM22180B | 3.51 | *Oncorhynchus mykiss* |
| CP012508.1 | PM32597B1 | 3.51 | *Oncorhynchus kisutch* |
| CP033937.1 | EM-90 | 3.69 | *Salmo salar* |
| CP061189.1 | Ps12201A | 3.65 | *Salmo salar* |
| CP048066.1 | Ps-8079A | 3.53 | *Salmo salar* |
| CP013944.1 | PSCGR01 | 3.49 | *Oncorhynchus mykiss* |
| CP038891.1 | Psal-005 | 3.32 | *Salmo salar* |
| CP039032.1 | Psal-070 | 3.35 | *Salmo salar* |
| CP039214.1 | Psal-104a | 3.45 | *Salmo salar* |
| CP038942.1 | Psal-027 | 3.45 | *Salmo salar* |
| CP038952.1 | Psal-040 | 3.48 | *Salmo salar* |
| CP038932.1 | Psal-025 | 3.52 | *Oncorhynchus mykiss* |
| CP039035.1 | Psal-071 | 3.45 | *Salmo salar* |
| CP038923.1 | Psal-011 | 3.42 | *Salmo salar* |
| CP038913.1 | Psal-010a | 3.40 | *Oncorhynchus kisutch* |
| CP038957.1 | Psal-041 | 3.43 | *Salmo salar* |
| CP038947.1 | Psal-028 | 3.47 | *Salmo salar* |
| CP038893.1 | Psal-006a | 3.47 | *Salmo salar* |
| CP039209.1 | Psal-103 | 3.44 | *Salmo salar* |
| CP038876.1 | Psal-002 | 3.43 | *Salmo salar* |
| CP038962.1 | Psal-051 | 3.45 | *Salmo salar* |
| CP038937.1 | Psal-026 | 3.39 | *Salmo salar* |
| CP013778.1 | PM51819A | 3.43 | *Salmo salar* |
| CP039040.1 | Psal-072 | 3.71 | *Salmo salar* |
| CP038972.1 | Psal-069 | 3.38 | *Salmo salar* |
| CP038881.1 | Psal-003 | 3.38 | *Oncorhynchus kisutch* |
| CP038886.1 | Psal-004 | 3.38 | *Salmo salar* |
| CP039204.1 | Psal-182 | 3.39 | *Salmo salar* |
| CP038811.1 | Psal-001 | 3.41 | *Oncorhynchus kisutch* |
| CP038967.1 | Psal-068 | 3.40 | *Salmo salar* |
| CP012413.1 | PM15972A1 | 3.25 | *Salmo salar* |
| CP039201.1 | Psal-163 | 3.30 | *Salmo salar* |
| CP039190.1 | Psal-160 | 3.34 | *Salmo salar* |
| CP013768.1 | PM23019A | 3.31 | *Salmo salar* |
| CP039186.1 | Psal-159 | 3.33 | *Salmo salar* |
| CP039181.1 | Psal-158 | 3.33 | *Salmo salar* |
| CP039065.1 | Psal-107 | 3.36 | *Oncorhynchus mykiss* |
| CP039195.1 | Psal-161 | 3.40 | *Salmo salar* |
| CP013773.1 | PM37984A | 3.29 | *Salmo salar* |
| CP013762.1 | PM21567A | 3.35 | *Salmo salar* |
| CP013757.1 | AY6492A | 3.35 | *Salmo salar* |
| CP013821.1 | PM25344B | 3.51 | *Oncorhynchus mykiss* |

**Supplementary Table 2**. Search for complete coding sequences and regions used in the LAMP assay of *ton*B-r, 1755 (Nitronate monooxygenase), and 1207 (HAD family acid phosphatase) genes for the identification and genotyping of P. salmonis in the NCBI database using the BlastN tool.

| **Gene name** | **Complete coding sequence** | | | | | **LAMP region** | | | | |
| --- | --- | --- | --- | --- | --- | --- | --- | --- | --- | --- |
| *ton*B-r | **Organism** | **Accession number** | **Identity (%)** | **E-value** | **Coverage (%)** | **Organism** | **Accession number** | **Identity (%)** | **E-value** | **Coverage (%)** |
|  | Bacterium SCSIO 12844 | CP073743.1 | 66.39 | 7e-85 | 59 | uncultured *Vibrio* sp. isolate | OY781095.1 | 68.23 | 4e-05 | 99 |
|  | *Vibrio tubiashii* strain FP17 | CP117029.1 | 66.58 | 7e-16 | 17 | *Vibrio* sp. STUT-A11 | AP026764.1 | 68.23 | 4e-05 | 99 |
|  | *Poseidonibacter lekithochrous* | CP054052.1 | 73.24 | 5e-36 | 12 | *Poseidonibacter lekithochrous* | CP054052.1 | 71.20 | 8e-14 | 96 |
|  | *Halarcobacter bivalviorum* strain LMG 26154 | CP031217.1 | 70.38 | 7e-22 | 12 | *Halarcobacter bivalviorum* strain LMG 26154 | CP031217.1 | 68.11 | 5e-04 | 95 |
|  | *Vibrio japonicus strain* JCM 31412 | CP102097.1 | 68.09 | 3e-13 | 12 | *Pseudomonas* sp. FLM 004-28 | OY817088.1 | 68.60 | 1e-04 | 90 |
| 1755 | Candidatus *Nitrososphaera evergladensis* SR1 | CP007174.1 | 76.92 | 5e-04 | 28 | *Bradyrhizobium* sp. 187 | CP064693.1 | 71.56 | 0.025 | 50 |
|  | *Bradyrhizobium* sp. | CP064693.1 | 71.56 | 0.006 | 18 | *Campaea margaritaria* | OU538792.1 | 89.74 | 0.025 | 17 |
|  | *Paenibacillus* sp. RC80 | CP137860.1 | 74.00 | 5e-04 | 17 | - | - | - | - | - |
|  | *Anoxybacillus caldiproteolyticus* strain U458 | CP064060.1 | 73.12 | 0.021 | 16 | - | - | - | - | - |
|  | *Clostridium* sp. JN-1 | CP033465.1 | 75.86 | 1e-04 | 15 | - | - | - | - | - |
| 1207 | *Coxiella burnetii* strain RSA439 | CP040059.1 | 65.64 | 5e-24 | 83 | *Francisella sp.* CA97-1460 | CP009654.1 | 78.38 | 5e-05 | 32 |
|  | *Coxiella burnetii str.* Schperling | CP014563.1 | 65.64 | 5e-24 | 83 | *Legionella oakridgensis* ATCC 33761 | CP004006.1 | 83.33 | 0.002 | 23 |
|  | *Francisella sp.* CA97-1460 | CP009654.1 | 76.09 | 4e-06 | 13 | - | - | - | - | - |
|  | *Legionella lytica* strain PCM 2298 | CP071527.1 | 76.83 | 2e-04 | 11 | - | - | - | - | - |
|  | *Legionella oakridgensis* ATCC 33761 | CP004006.1 | 83.05 | 2e-04 | 8 | - | - | - | - | - |
